# A Multi‐factor Analysis of Revision in Total Hip Replacement Using the Collarless‐Polished‐Tapered Stems with Different Cups

**DOI:** 10.1111/os.13778

**Published:** 2023-06-29

**Authors:** Yu Hong, Linda Johnston, Weijie Wang

**Affiliations:** ^1^ University Department of Orthopaedic and Trauma Surgery Ninewells Hospital and Medical School, TORT Centre, University of Dundee Dundee UK

**Keywords:** Implant combination, Multi‐factors, Revision, The CPT implant, The Trilogy implant, Total hip replacement

## Abstract

**Objective:**

Collarless‐polished‐tapered (CPT) stems have been widely used in total hip replacement (THR). Given that various types of cups are combined with CPT in clinical practice, however, what cup type performs the best for use with CPT is still unclear. This study aimed to investigate the effects of three types of commonly used cups with CPT on revision and survival life using multi‐factor analysis.

**Methods:**

This study is a cohort study using the data between October 1998 to September 2021. The data of THR patients with ZCA All‐poly Acetabular Cup, Continuum Acetabular System, and Trilogy Acetabular System with CPT were collected from several hospitals in the UK. The patients aged from 20 to 97 (n = 5981, 2345 male and 3636 female). Age, gender, body mass index, diagnosis, surgeon grade, cup material, cup size, surgical approach, survival life, complications, and Harris hip scores (HHS) were analyzed in relation to revision status. SPSS statistical software was used to analyze the relationship among various factors. The main statistical methods included chi‐square with cross tables, analysis of variance (ANOVA) and survival analysis.

**Results:**

The results in relation to HHS shows that the continuum cup has the best outcome in the postoperative period of 1 and 5 years (1 year = 90.7, 5 years = 91.3; *P* < 0.001); the Trilogy cup was the second (1 year = 88.4, 5 years = 87.3; *P* < 0.001); and the ZCA cup was the third (1 year = 84.6, 5 years = 82.4; *P* < 0.001). However, the Trilogy cup performed the best regarding survival life on revision while the Continuum cup was the worst.

**Conclusion:**

When the CPT stem is combined with different cups, the trilogy cup shows the best characteristics in terms of survival trends with revision ratios compared with the continuum and ZCA cups, and is therefore recommended by this study.

## Graphical abstract

Three cups with CPT were investigated in the 5981 total hip replacement patients. It is found that the trilogy type has the best performance in terms of survival life on revision.

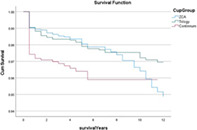

## Introduction

The incidence of osteoarthritis (OA) is increasing with the ever‐increasing age of the global population and increasing obesity rate.^1^, ^2^ It is estimated that 250 million people worldwide are affected by this complex syndrome.^3^ Osteoarthritis is a disease that can affect almost every joint.^4^ Hunter and Bierma‐Zeinstra and Zaki *et al*. also present that pathological changes in cartilage, bone, synovium, ligament, muscle, and fat present around joints leads to a series of symptoms including joint dysfunction and pain, stiffness, and limited function, among others.^3^, ^5^ The etiology of OA remains unclear, but it is generally believed that the main risk factors of OA are age, being female, obesity, heredity and serious common injuries.^6^ Three percent of people over 75 years of age have symptoms and imaging manifestations of OA of the knee joint.^7^ In the 1960s, total hip replacement (THR) was used to treat patients with OA, which completely changed the previous treatment methods and effectively improved the prognosis of patients.^8^, ^9^, ^10^, ^11^ Although THR is a common surgical operation in which implants replace diseased joints and restore normal hip function and quality of life, revision surgery still exists. Therefore, the reasons for the revision should be further studied.^12^

Although Pakarinen *et al*.^13^ reported that the collarless‐polished‐tapered (CPT) implant has higher revision rates compared to Exeter implant due to periprosthetic femoral fracture, CPT remains one of the most used types in hospitals according to the Tayside Arthroplasty Audit Group (TAAG) database, Scotland. Further, although many types of cups have been used with CPT currently in hospitals, there is little research available on which cup combined with CPT could provide better or best outcomes for THR patients.^14^ Therefore, this study aimed to compare three commonly used cups (ZCA All‐poly Acetabular Cup, Continuum Acetabular System and Trilogy Acetabular System combined with CPT) to investigate which cup is better suited for CPT in terms of revision ratios, hip scores, and implant survival. The study analyzed the relationships between multi‐factor, demographically and clinically with outcomes in THR, and thus provided clinical guidance and suggestions. Therefore, the purposes of this study were: (i) to compare three commonly used cups (ZCA All‐poly Acetabular Cup, Continuum Acetabular System and Trilogy Acetabular System combined with CPT) to investigate which cup is better suited for CPT in terms of revision ratios, hip scores, and survival functions, and (ii) to analyze the relationships among the multi‐factor, demographically and clinically with outcomes in THR, and thus provided clinical guidance and suggestions.

## Materials and Methods

### Literature Review

The primary search was done from University Library Electronic Journals, PubMed, Scopus, Google Scholar, Science Direct and Web of Science. The terms used in the search were “total hip arthroplasty,” “total hip replacement,” “total hip replacement in the UK,” “Harris Hip Scores,” “modified Harris Hip Scores,” “compare prosthesis brands in the UK,” “compare the cup prostheses in the UK,” “the postoperative outcome of CPT in total hip replacement,” “ZCA cup,” “Continuum cup,” “Trilogy cup.” The previously published studies and reports relevant to THR cases and also containing observational studies and random controlled trials were collected. Moreover, these articles related to the CPT from Zimmer Biomet, the cups ZCA, Continuum, and Trilogy from Zimmer Biomet were reviewed. All the references were in the English language, and to gain updated progress, articles published since 2005 were considered.

### The Characteristics of Cup Implants–ZCA, Continuum and Trilogy

**Fig. 1:**
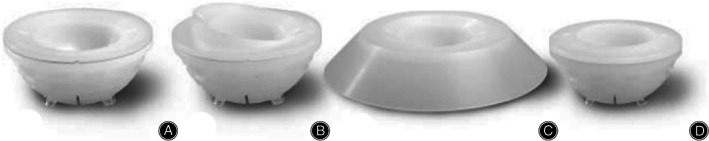
The 4 styles of ZCA cups: (A) Neutral Cup, (B) Inclined Face Cup, (C) Flanged Cup, and (D) Snap‐In Cup. Available at: https://orto.hi.is/skrar/zca_all_poly_acetabu549.pdf (Accessed date: 24 June 2022).

**Fig. 2:**
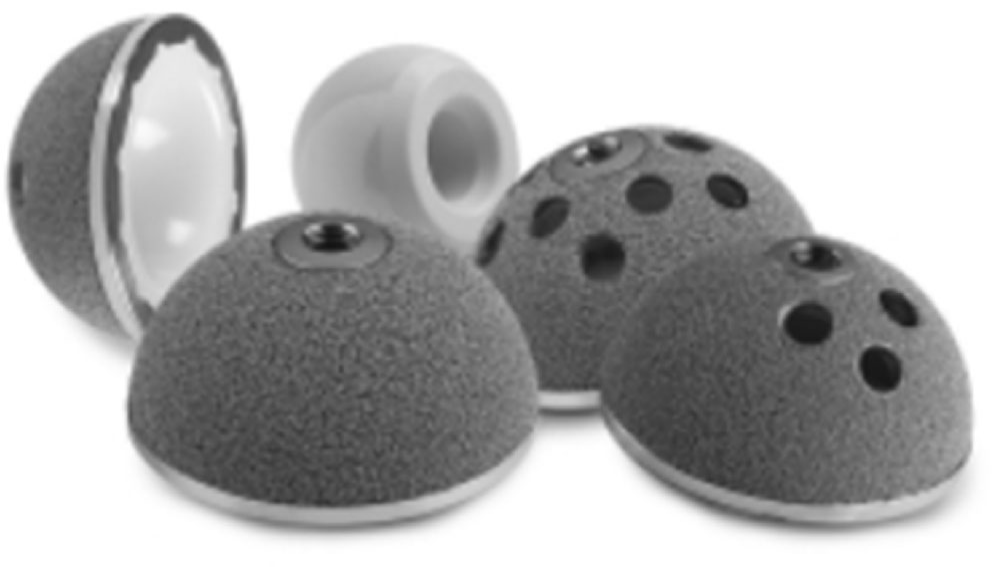
Continuum® Acetabular System. Available at: https://www.zimmerbiomet.lat/en/medical‐professionals/hip/product/continuum‐acetabular‐system.html (Accessed date: 24 June 2022).

**Fig. 3:**
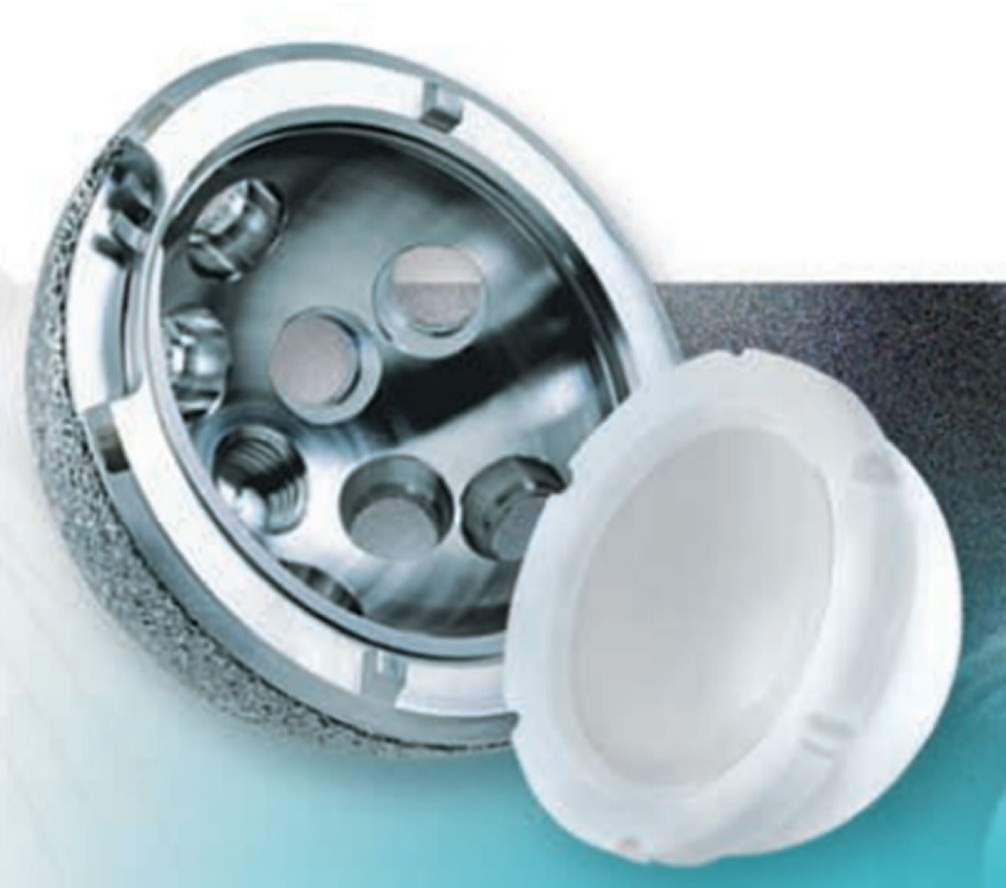
Trilogy acetabular Hip system. Available at: https://www.zimmerbiomet.lat/en/medical‐professionals/hip/product/trilogy‐acetabular‐hip.html (Accessed date: 24 June 2022).

The ZCA cup implant is an all‐poly acetabular cup that uses cemented fixation and has four styles of cup (Fig. 1); the material of the ZCA cup is polyethene, and it has a maximum thickness of 6 mm, and each has four 3 mm cement spacers. A previous study found that the conventional polyethene version of the ZCA cup had a similar revision risk to the highly crosslinked polyethene version (*P* = 0.09)^14^ but the TAAG database did not provide the data on the later version. The Continuum cup is a highly porous trabecular metal, and the clinical history is over 19 years (Fig. 2). Furthermore, the Vitamin E, Highly Crosslinked Polyethylene, Longevity® Highly Crosslinked Polyethylene and BIOLOX® delta Ceramic Technology could increase wear resistance of this implant. However, the Continuum has a higher risk of revision due to dislocation.^15^ The third type of cup implant is the Trilogy design, based on the Harris‐GalanteTM Porous and HGP II Cups, can inhibit the formation and migration of polyethene debris (Fig. 3). In addition, this type of cup implant liner is also made with Longevity Highly Crosslinked Polyethylene and BIOLOX® delta Ceramic Technology and means that the Trilogy cup has very low wear and high fracture resistance.

### Data Extraction

The data were collected from the Tayside Arthroplasty Audit Group (TAAG) database which contains data from several hospitals in the local area. The TAAG database follows‐up patients for their lifetime where possible. THR case data ranged from October 1, 1998 to September 9, 2021. Each case includes information on leg/side, age, gender, body mass index (BMI), diagnosis, surgeon, material of cup implant, cup size, surgical approach, length of hospital stay, survival years, Harris hip scores (HHS), modified Harris hip scores (mHHS), revision rate and complication groups of every patient. The data used included 5981 CPT stem cases of which 2697 cases used a ZCA cup, 1964 cases used the Trilogy cup, and 1320 cases used Continuum cup. To access the data, Caldicott Guardian Approval was granted from the School of Medicine Research Ethical Committee, the University of Dundee (No. SC015096).

### Grouping

The data for each factor were grouped according to commonly used standards (Table 1).

**TABLE 1:** Data grouping

Variables	Grouping
Cup	ZCA
	Continuum
	Trilogy
Leg	Left
	Right
Age	<60
	>=60
Gender	Male
	Female
BMI	<18.5
	18.5–24.9
	25–29.9
	>=30
Diagnosis	Osteoarthritis
	Fracture
	Others
Surgeon	Consultant
	Trainee
Cup materials	Ceramic
	Cobalt chrome
	Stainless steel
Cup size	<28 mm
	28–32 mm
	>32 mm
Surgical approach	Anterolateral
	Posterior
	Others
Harris hip score & modified Harris hip score	<70
	70–79
	80–89
	>=90

### Statistical Methods

This study used SPSS Statistical version 28 software (IBM, Armonk, NY, USA) to analyze the local data. The three functions used were cross tables, analysis of variance (ANOVA), and survival analysis. To describe single categorical variables the frequency table was used, and to describe the relationship between two categorical variables the cross tables was used, for example, between gender and revision. The second function is the one‐way ANOVA, which was used to determine whether there were any statistically significant differences between the means of two or more independent or unrelated groups for numeric variables, for example, HHS. The last function used in this study was survival analysis, which analyzed the ratios of the number of cases where a revision occurred as a terminal sign to the number of cases for the whole population in a specific period with time progressing. Whole revision ratios were calculated as the proportion of the number of total cases with revision to the number of total cases collected. However, after a postoperative period of 10 years, the Continuum cup only had a case, so the time interval of the survival analysis for it must end at a postoperative period of 10 years.

In short, this study used cross tables to describe the multi‐factors within the cup groups and calculated the chi‐square to analyze which factors had statistical significance; then used the one‐way ANOVA to compare the HHS and mHHS in different following years, and then compared which implant combinations had good outcome and which factors were effective for the prosthesis by survival analysis.

## Results

### Basic Information

Table 2 highlights the mean ± standard of age, BMI, head size and length of stay (LenStay), and the reported range. All the *P*‐values from Table 2 were less than 0.05, which means these results had statistical significance. Most of the variables were regrouped in this study and these results are shown in Tables 3 and 4. It is noted that except for the Leg group, all the other variables were *P* < 0.05. As whole, various cup groups were not significantly differ in revision rates as seen in Table 5. However, there were some significant differences in terms of different factors.

**TABLE 2:** Basic information for age, BMI, head size, gender and length of stay

Variable	Cup	N	Mean	Std. deviation	Minimum	Maximum
Age (year)	ZCA	2697	74.4	8.1	26	97
	Trilogy	1964	66.1	9.1	20	97
	Continnum	1320	61.0	10.0	21	91
BMI	ZCA	2524	28.1	5.1	13.5	71.7
	Trilogy	1813	28.8	5.2	11.6	64.9
	Continnum	1239	29.3	5.7	11.3	64.8
Head size (mm)	ZCA	2288	28.8	2.5	22	32
	Trilogy	1541	29.2	2.8	22	40
	Continnum	1299	32.2	3.1	28	40
Length stay (day)	ZCA	2696	6.1	4.7	0	69
	Trilogy	1961	5.7	3.7	0	69
	Continnum	1319	3.9	3.4	0	53

*Note*: For each group, *P* < 0.01.

**TABLE 3:** Comparison of the patients' information (n and %) within the cup group

		Cup group			Total
Variable	Cup	ZCA	Trilogy	Continnum	
Leg group	Left	1178 (43.7%)	877 (44.7%)	614 (46.5%)	2669
	Right	1519 (56.3%)	1086 (55.3%)	706 (53.5%)	3311
Total		2697	1963	1320	5980
Age group	<60	109 (4.0%)	369 (18.8%)	546 (41.4%)	1024
	≥60	2588 (96.0%)	1595 (81.2%)	774 (58.6%)	4957
Total		2697	1964	1320	5981
BMI group	<18.5	39 (1.5%)	16 (0.9%)	9 (0.7%)	64
	18.5–24.9	659 (26.1%)	419 (23.1%)	264 (21.3%)	1342
	25–30	1050 (41.6%)	721 (39.8%)	461 (37.2%)	2232
	>30	776 (30.7%)	657 (36.2%)	505 (40.8%)	1938
Total		2524	1813	1239	5576
Gender group	Male	905 (33.6%)	865 (44.0%)	575 (43.6%)	2345
	Female	1792 (66.4%)	1099 (56.0%)	745 (56.4%)	3636
Total		2697	1964	1320	5981

*Note:* The *p*‐value of Leg group was 0.236, and the other groups *P*‐values < 0.01.

**TABLE 4:** Comparison of the clinical information (n and %) within the cup group

		Cup group			Total
Variable	Cup	ZCA	Trilogy	Continnum	
Approach group	Anterolateral	1446 (53.8%)	1496 (76.3%)	485 (36.8%)	3427 (57.5%)
	Posterior	940 (35.0%)	345 (17.6%)	819 (62.1%)	2104 (35.3%)
	Others	300 (11.2%)	119 (6.1%)	14 (1.1%)	433 (7.3%)
Total		2686	1960	1318	5964
Head size group	<28	145 (6.3%)	16 (1.0%)	0 (0.0%)	161 (3.1%)
	28–32	2143 (93.7%)	1408 (91.4%)	1003 (77.2%)	4554 (88.8%)
	>32	0 (0.0%)	117 (7.6%)	296 (22.8%)	413 (8.1%)
Total		2288	1541	1299	5128
Head group	Ceramic	71 (2.6%)	392 (20.1%)	762 (57.9%)	1225 (20.6%)
	Cobalt Chrome	1937 (72.3%)	844 (43.3%)	554 (42.1%)	3335 (56.1%)
	Stainless Steel	672 (25.1%)	712 (36.6%)	0 (0.0%)	1384 (23.3%)
Total		2680	1948	1316	5944
Surgeon group	Consultant	1823 (67.6%)	1572 (80.0%)	1044 (79.1%)	4439 (74.2%)
	Trainee	874 (32.4%)	392 (20.0%)	276 (20.9%)	1542 (25.8%)
Total		2697	1964	1320	5981
Diagnosis group	Osteoarthritis	2412 (89.9%)	1869 (95.3%)	1088 (82.9%)	5369 (90.1%)
	Fracture	204 (7.6%)	31 (1.6%)	145 (11.0%)	380 (6.4%)
	Others	66 (2.5%)	61 (3.1%)	80 (6.1%)	207 (3.5%)
Total		2682	1961	1313	5956

*Note*: For each group, the *P*‐value results are <0.001.

**TABLE 5:** Summary of revision rates for all cup groups

		Revision		
Variable	Cup	Yes	No	Total
ZCA	Count	67	2630	2697
	% within group	2.5%	97.5%	100.0%
Trilogy	Count	55	1909	1964
	% within group	2.8%	97.2%	100.0%
Continnum	Count	44	1276	1320
	% within group	3.3%	96.7%	100.0%
Total	Count	166	5815	5981
	% within group	2.8%	97.2%	100.0%

*Note*: *P* < 0.305 by chi‐square test using SPSS crosstab function.

### Harris Hip Scores

**Fig. 4:**
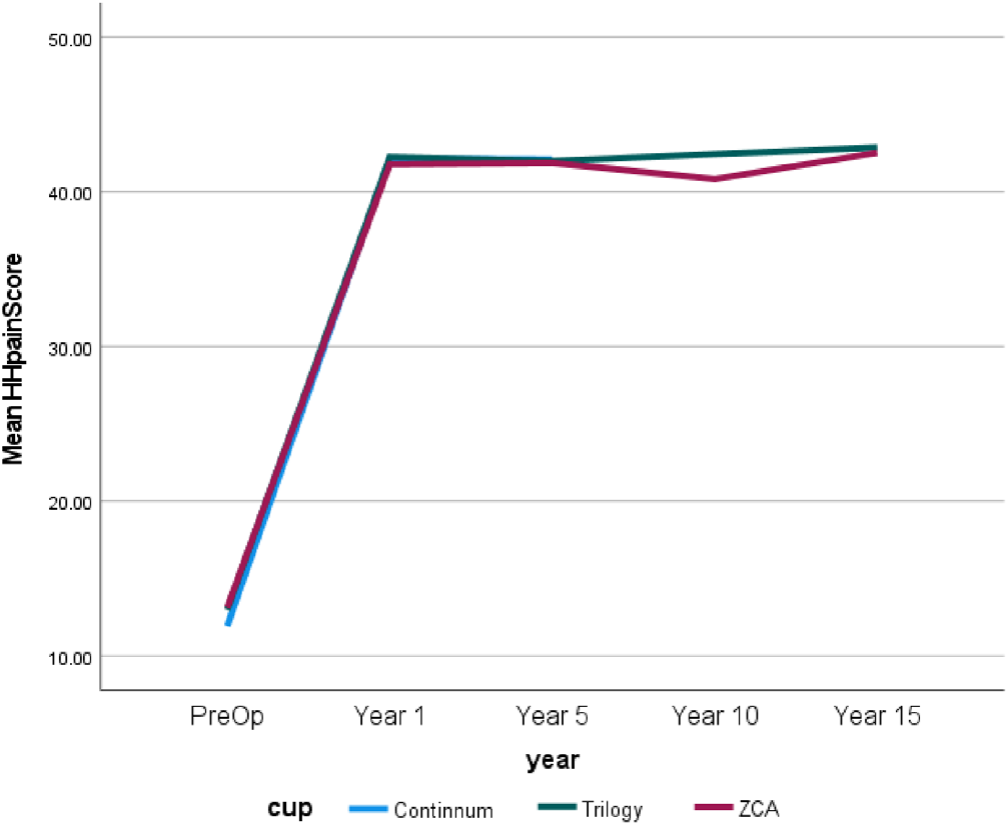
Line chart compares Harris hip pain score between the preoperative and postoperative.

**Fig. 5:**
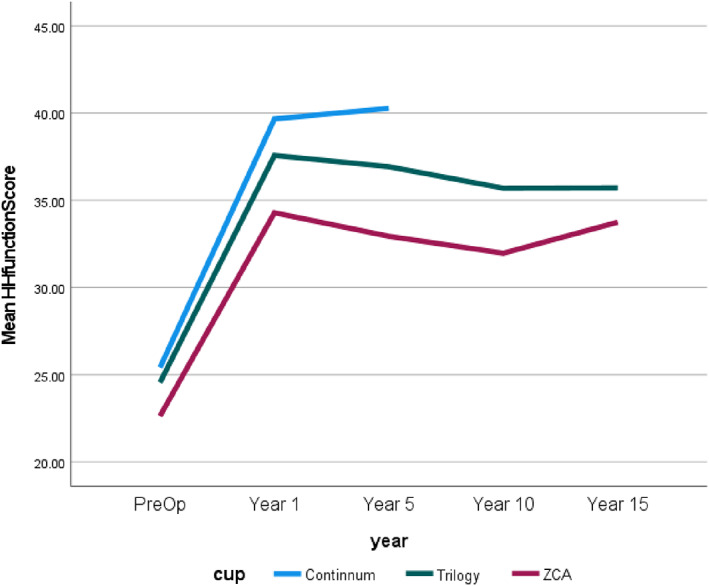
Line chart compares the Harris hip function score between the preoperative and postoperative.

**Fig. 6:**
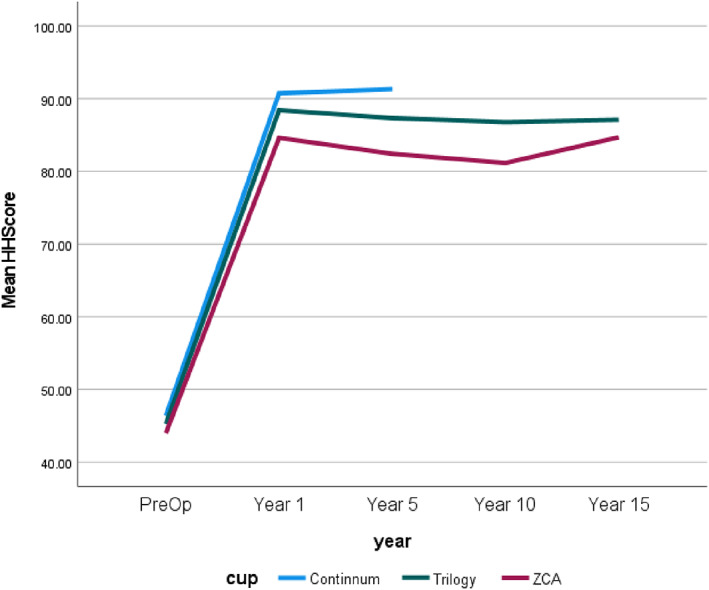
Line chart compares the Harris Hip Score between the preoperative and postoperative.

**Fig. 7:**
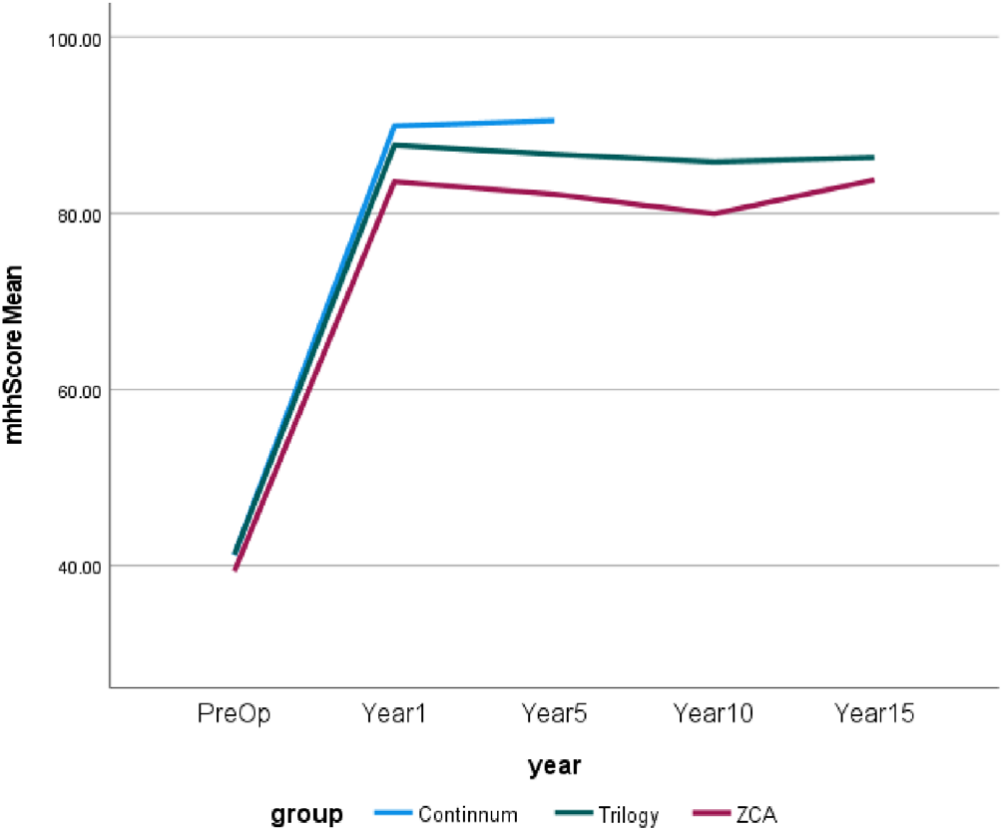
Line chart compares the modified Harris Hip Score between the preoperative and postoperative.

It should be noted that though there were 1320 samples for the Continuum cup, HH scores covered pre‐operative to 5 years. For convenience and to show the results of the HHS, Figs 4, 5, 6, 7 simply present the line trends of the HHS results within the Cup Group. Figure 4 illustrates HHS pain scores where the Continuum cup performed the best at postoperative in years 1 and 5, was similar to the Trilogy cup. It was noted that only the *P*‐value of the postoperative 1 year had statistical significance in Harris Hip Pain Scores. Figure 5 also shows the same results as Fig. 4, the Continuum implant performed well at postoperative year 1 and years 5, the Trilogy and the ZCA followed. The *P*‐value at postoperative 1 and 5 years were significant. In addition, Fig. 6 reported that only the Continuum cup results at postoperative 1 year and 5 years were over 90 in the HHS results, which could be defined as excellent results. The result of the Trilogy was still similar with the Continuum implant, and the *P*‐value at postoperative 1 year and 5 years was less than 0.05, so these results are significant. Furthermore, the mHHS and *P*‐value results were similar to the HHS results but only the Continuum cup results at postoperative 5 years were greater than 90. In short, the Continuum implant performed best, followed by the Trilogy implant. Due to the lack of Continuum data after 10 years, the further comparison is impossible.

### Compare the Cup Group by Survival Years

**Fig. 8:**
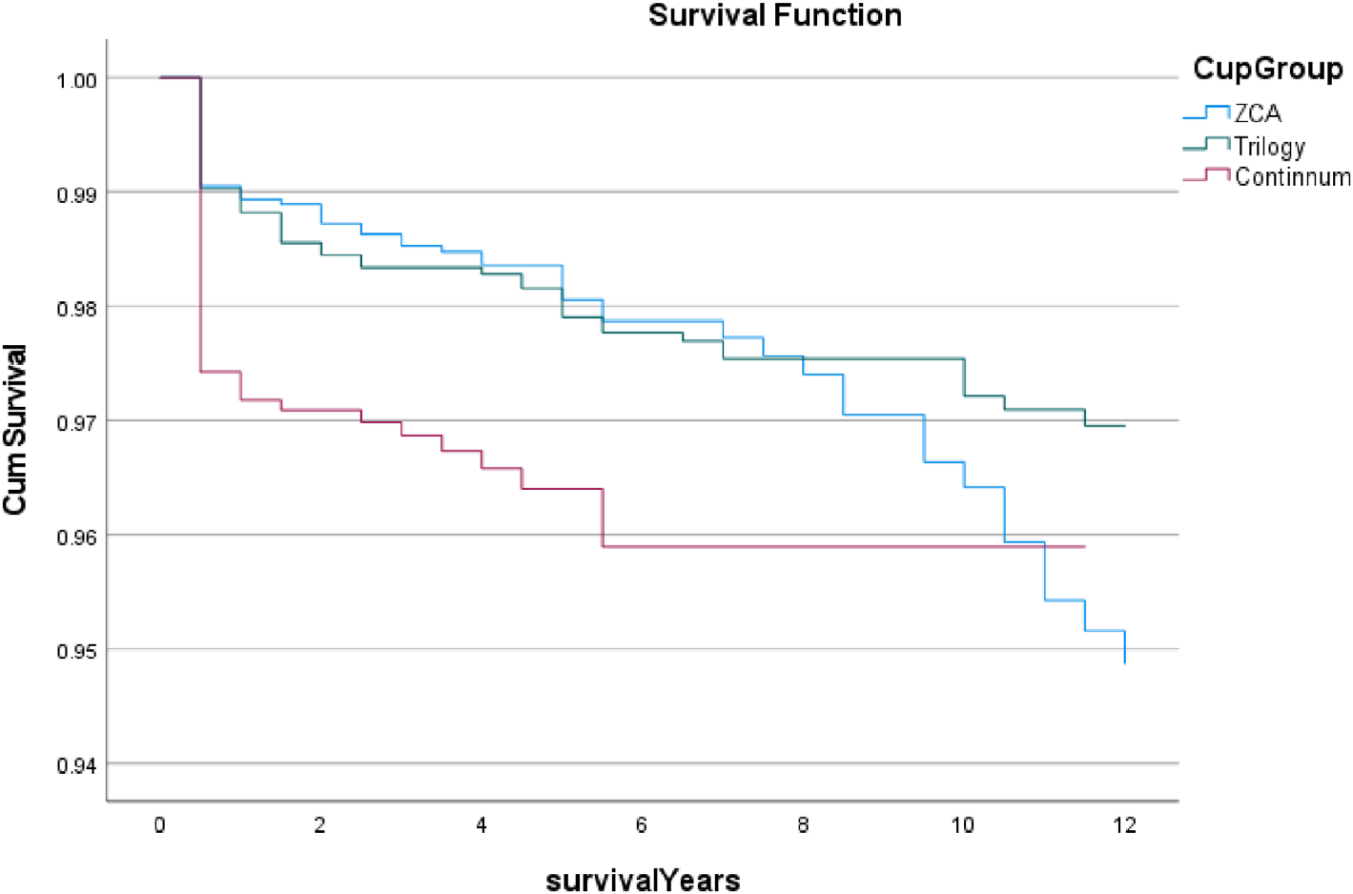
The survival rates for revision between cup groups within 12 years. Note: Both ZCA and Trilogy had a median 12 years survival period, longer than Continuum which had 11 years (*p* < 0.004). * The *p*‐value of overall comparisons is <0.001.

As most of the survival data for three cups covered year 1 to 12, the survival analysis was carried out within 12 years. Figure 8 reports the survival rates of revision between cup groups within 12 years. It was found that different cup groups had a huge difference at postoperative 1 year, the lines of the ZCA and Trilogy cups are interwoven until postoperative 9 years and the line of the Trilogy cup is approximately a straight line, but the ZCA drops rapidly. It is noted that the Continuum cup line drops quickly at postoperative 1 year. As a whole, both ZCA and Trilogy cups had a median survival period of 12 years which was better than the Continuum cup at 11 years (*P* < 0.004).

Table 6 shows the complication ratios between the cup groups as whole data. It is obvious that the Trilogy cup is slightly better than the other cup groups in orthopedic‐related cases, and the Continuum cup is better in “No” complication ratio (*P* < 0.001).

**TABLE 6:** Summary of complication rates for all cup groups

Variable	Cup group			Total
Complications	ZCA	Trilogy	Continnum	
Orthopedic‐related	248	172	118	538
	9.2%	8.8%	8.9%	9.0%
None orthopedic‐related	197	89	51	337
	7.3%	4.5%	3.9%	5.6%
No	2252	1703	1151	5106
	83.5%	86.7%	87.2%	85.4%
Total	2697	1964	1320	5981
	100.0%	100.0%	100.0%	100.0%

*Note*: *P* < 0.001 by chi‐square test.

In general, the results reported that the Continuum cup performed the best in relation to HHS within follow‐up 5 years, but the Trilogy cup was better in relation to revision and survival life. Moreover, Tables 3 and 4 reported that except for the leg factor, the other factors would affect the outcome of THR. Thus, this research continues to analyze which factors could decrease the revision rates within use the CPT combined with the Trilogy and would have a good prognosis.

**Fig. 9:**
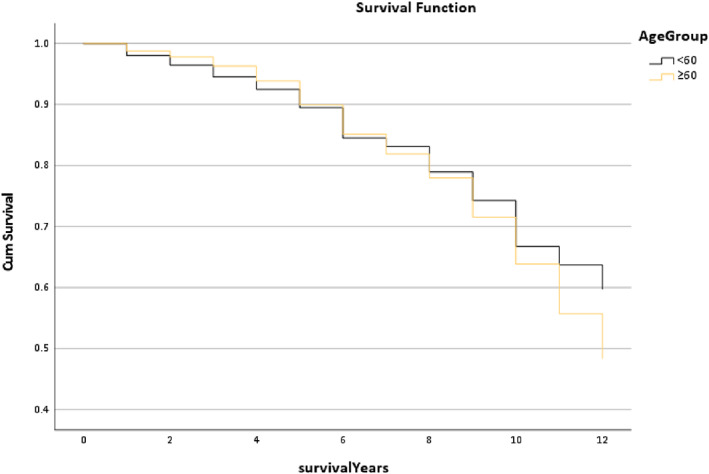
The survival rate for the age group in the Trilogy group within 12 years. Note: The median survival time of the <60 group (12 years) was similar to the > = 60 group (11.78 years). * The *P*‐value of overall comparisons is 0.294.

Figure 9 presents the survival rate of the Age Group for the Trilogy cup. The line trends of these groups still interweave from the preoperative to the postoperative 12 years. The median survival time of the <60 group (12 years) was similar to the > = 60 group (11.78 years), but the overall *P*‐value is greater than 0.05 meaning this result did not have statistical significance.

**Fig. 10:**
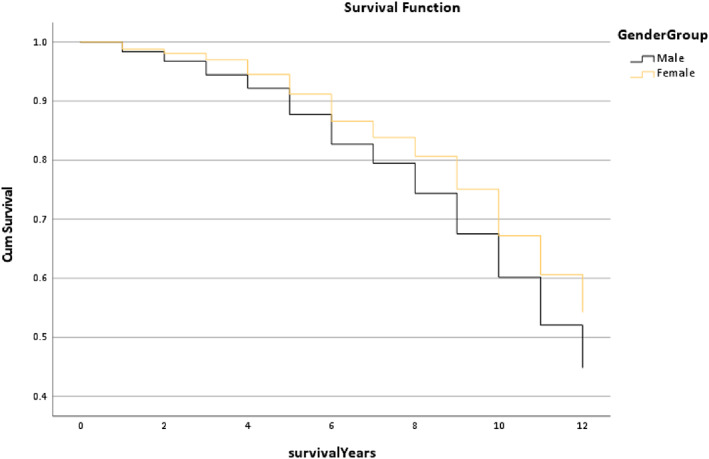
The survival rate for the gender group in the Trilogy group within 12 years. Note: The median survival time of the Female group was 12 years which was better than the Male group (11.29 years). * The *P*‐value of overall comparisons is <0.001.

Figure 10 shows the survival rate of the Gender Group and shows the line trend of the Female Group was slightly higher than the Male Group. Moreover, the median survival time of the Female Group was 12 years which was better than the Male Group (11.29 years) (*P* < 0.001).

**Fig. 11:**
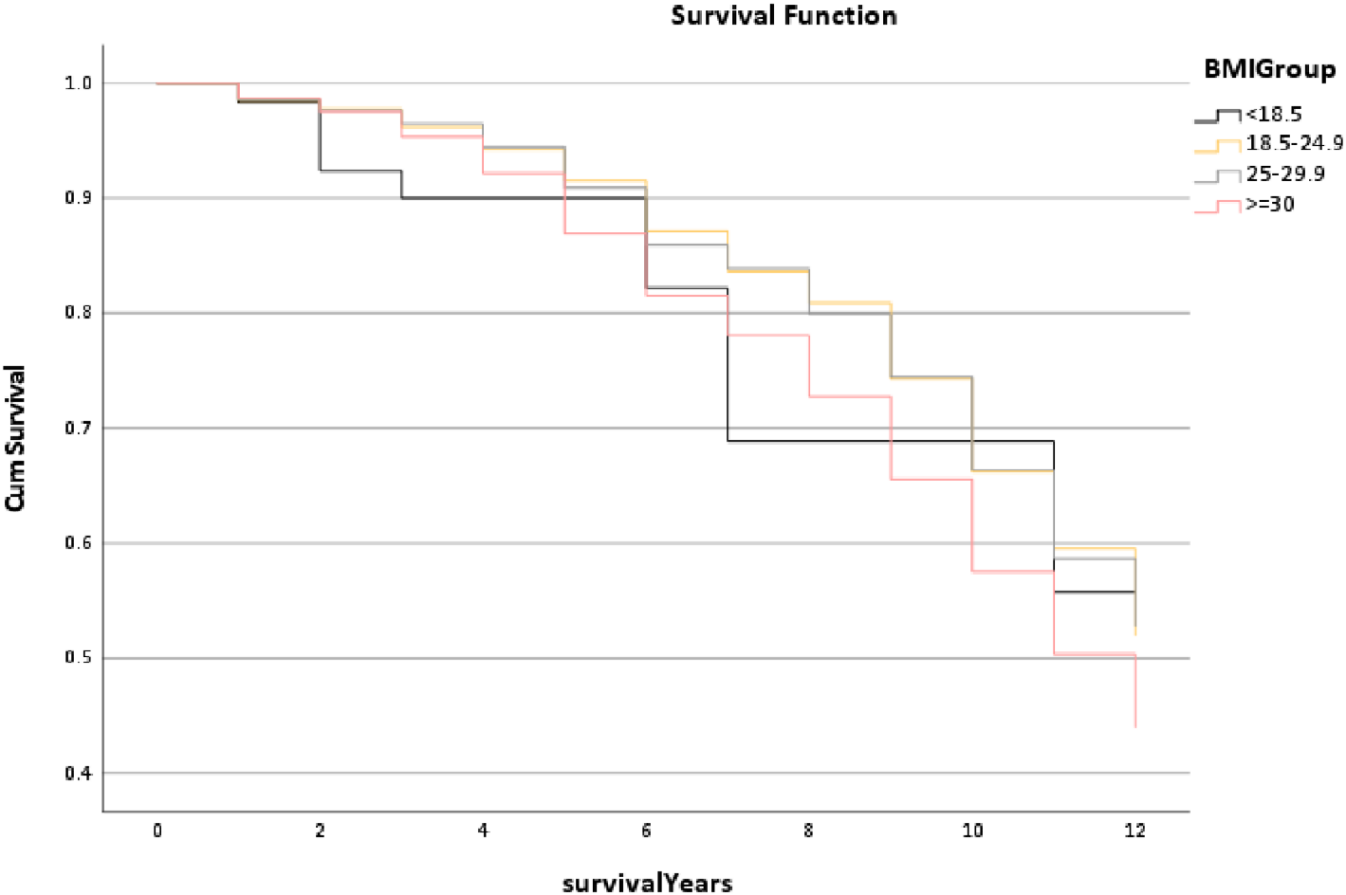
The survival rate for the BMI group in the Trilogy group within 12 years. Note: Except for the median survival time of the > = 30 group was 11.06 years, the other groups were the same (12 years). * The *P*‐value of overall comparisons is <0.001.

Figure 11 reports the survival rate of the BMI Group in the Trilogy cup, and the line trends have differences. It found that the <18.5 group line was lower than the other lines, especially at postoperative 3 years and 7 years, but the line trends of the 18.5–24.9 and the 25–29.9 groups were interwoven closely. Furthermore, except for the median survival time of the > = 30 group was 11.06 years, the other groups were the same (12 years) (*P* < 0.001).

**Fig. 12:**
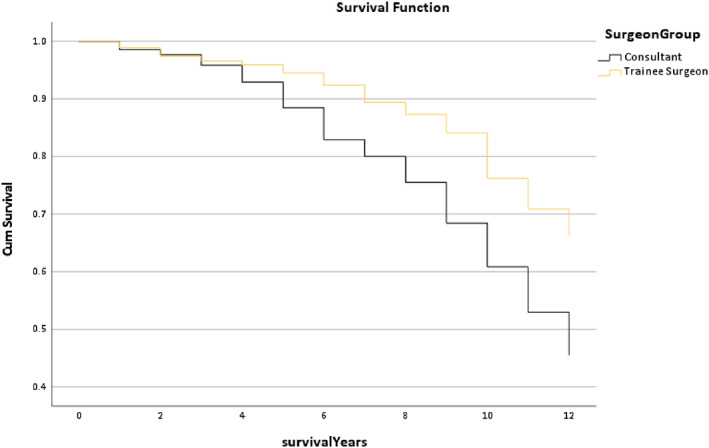
The survival rate for the surgeon group in the Trilogy group within 12 years. Note: The median survival time of the Trainee Surgeon was 12 years which was better than the Consultant (11.40 years). * The *P*‐value of overall comparisons is <0.001.

Figure 12 shows the survival rate of the Surgeon Grade Group in the Trilogy cup within 12 years. It is noted that the line of the Trainee Surgeon Group was higher than the Consultant Group after postoperative 4 years, and the median survival time of the Trainee Surgeon was 12 years which was better than the Consultant (11.40 years) (*P* < 0.001).

**Fig. 13:**
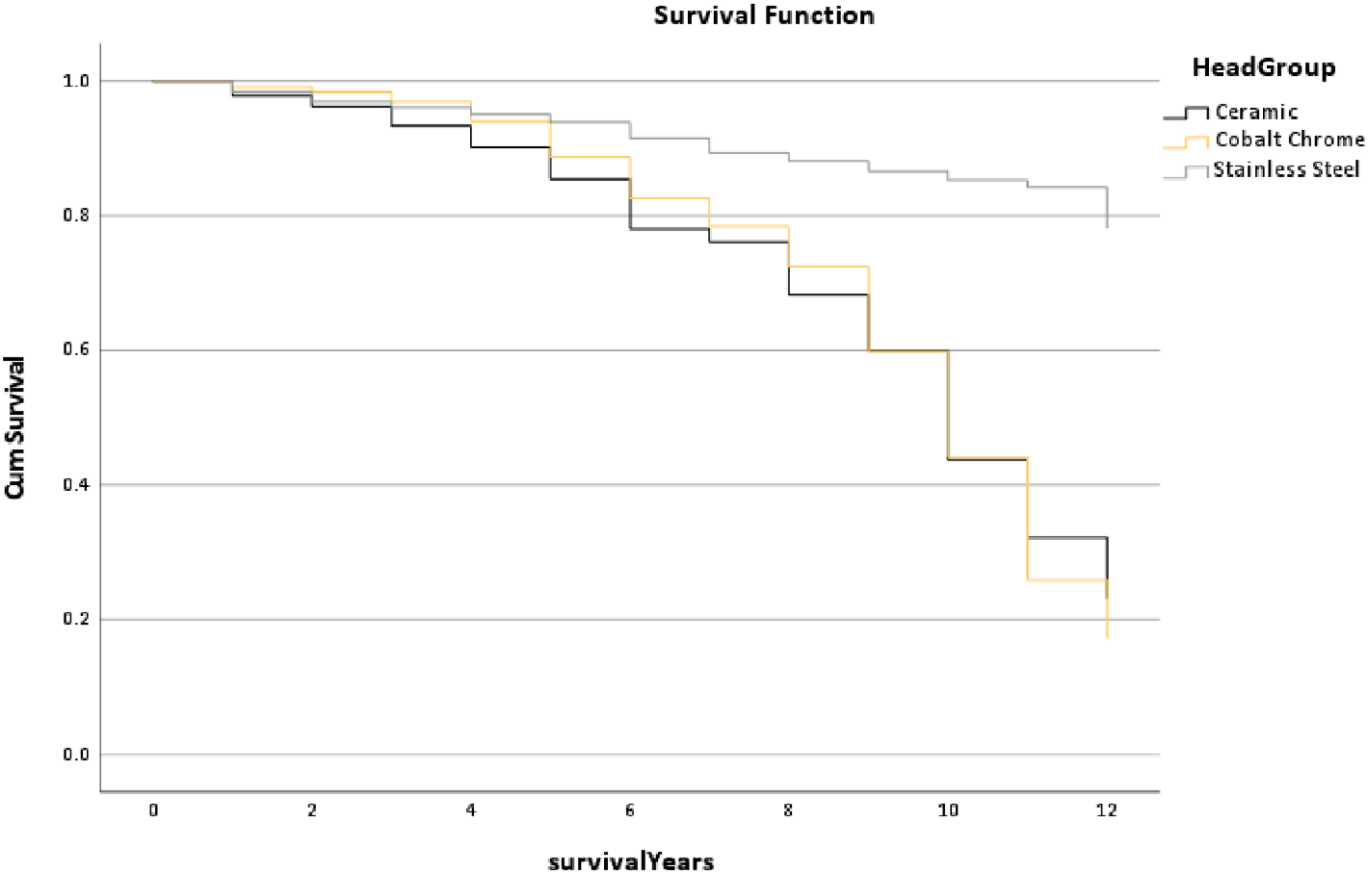
The survival rate for the head material group in the Trilogy group within 12 years. Note: The median survival time of the ceramic and cobalt chrome groups were similar, which were 9.62 years and 9.63 years. But the stainless steel has 12 years. * The *P*‐value of overall comparisons is <0.001.

Figure 13 reports the survival rate of the Head Materials Group and it is noted that the Stainless line tends to be a straight line within 12 years, but the line trends of the other groups are interwoven. In addition, the median survival time of the Ceramic and Cobalt Chrome groups were similar, being 9.62 years and 9.63 years, respectively with the Stainless‐Steel being 12 years (*P* < 0.001).

**Fig. 14:**
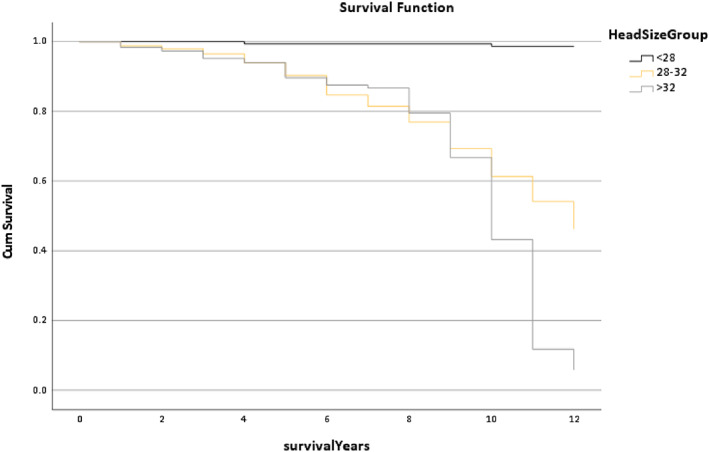
The survival rate for the head size group in the Trilogy group within 12 years. Note: The median survival time of these two groups was similar, which was 12 years and 11.53 years, as the >32 mm Group only has 9.71 years. * The *P*‐value of overall comparisons is <0.001.

Figure 14 shows the survival rate of the Head Size Group. This found that the line trend of the <28 mm Group almost performed as a straight line, but the line trend of the 28 to 32 mm, which was the most commonly used group, declined. The median survival time of these two groups was similar at 12 years and 11.53 years, respectively, as the >32 mm Group was only 9.71 years (*P* < 0.001).

**Fig. 15:**
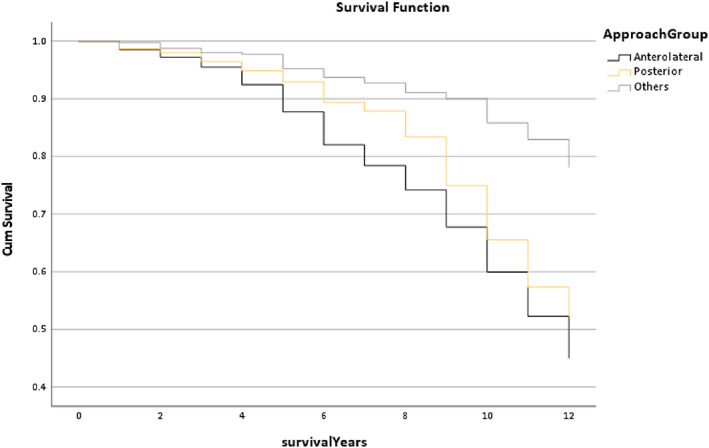
The survival rate for the Approach group in the Trilogy group within 12 years. Note: The median survival time of the Posterior and the Others groups was 12 years, but the Anterolateral Group only has 11.31 years. * The *P*‐value of overall comparisons is <0.001.

Figure 15 presents the survival rate of the Approach Group and it found that the line trend of the Others group—this group contains all the other surgical approaches—was higher than the other groups. Moreover, the median survival time of the Posterior and the Others groups was 12 years, but the Anterolateral Group was only 11.31 years (*P* < 0.001).

In the database used, out of all cases, 11 cases were linked to osteoporosis and none of them was linked to revision. Therefore, it is unlikely that osteoporosis leads to revision. To search the reasons for the revision, we also compared three groups in terms of complications. The result showed that ZCA was the highest rate in complications and other two had similar rates (*P* < 0.001) as seen in Table 7.

**TABLE 7:** Complication rates in three cup groups

		Cup group			Total
Variable	Cup	ZCA	Trilogy	Continnum	
Wound drainage	Count	17	2	1	20
	% within CupGroup	0.6%	0.1%	0.1%	0.3%
	% of Total	0.3%	0.0%	0.0%	0.3%
Urinary tract infection	Count	24	5	14	43
	% within CupGroup	0.9%	0.3%	1.1%	0.7%
	% of Total	0.4%	0.1%	0.2%	0.7%
Swollen limb	Count	4	3	0	7
	% within CupGroup	0.1%	0.2%	0.0%	0.1%
	% of Total	0.1%	0.1%	0.0%	0.1%
Superficial infection	Count	13	19	13	45
	% within CupGroup	0.5%	1.0%	1.0%	0.8%
	% of Total	0.2%	0.3%	0.2%	0.8%
Subluxation	Count	4	0	1	5
	% within CupGroup	0.1%	0.0%	0.1%	0.1%
	% of Total	0.1%	0.0%	0.0%	0.1%
Pulmonary oedema	Count	10	8	1	19
	% within CupGroup	0.4%	0.4%	0.1%	0.3%
	% of Total	0.2%	0.1%	0.0%	0.3%
Pressure sores	Count	9	1	0	10
	% within CupGroup	0.3%	0.1%	0.0%	0.2%
	% of Total	0.2%	0.0%	0.0%	0.2%
Peri‐prosthetic fracture	Count	18	30	13	61
	% within CupGroup	0.7%	1.5%	1.0%	1.0%
	% of Total	0.3%	0.5%	0.2%	1.0%
Peri‐operative fracture	Count	29	9	4	42
	% within CupGroup	1.1%	0.5%	0.3%	0.7%
	% of Total	0.5%	0.2%	0.1%	0.7%
Nerve injury	Count	7	6	2	15
	% within CupGroup	0.3%	0.3%	0.2%	0.3%
	% of Total	0.1%	0.1%	0.0%	0.3%
Myocardial infarction	Count	4	5	2	11
	% within CupGroup	0.1%	0.3%	0.2%	0.2%
	% of Total	0.1%	0.1%	0.0%	0.2%
Haematoma	Count	11	8	9	28
	% within CupGroup	0.4%	0.4%	0.7%	0.5%
	% of Total	0.2%	0.1%	0.2%	0.5%
Gastrointestinal	Count	14	6	1	21
	% within CupGroup	0.5%	0.3%	0.1%	0.4%
	% of Total	0.2%	0.1%	0.0%	0.4%
Excessive related	Count	7	5	3	15
	% within CupGroup	0.3%	0.3%	0.2%	0.3%
	% of Total	0.1%	0.1%	0.1%	0.3%
Dislocation	Count	58	48	30	136
	% within CupGroup	2.2%	2.4%	2.3%	2.3%
	% of Total	1.0%	0.8%	0.5%	2.3%
Deep vein thrombosis	Count	12	6	6	24
	% within CupGroup	0.4%	0.3%	0.5%	0.4%
	% of Total	0.2%	0.1%	0.1%	0.4%
Deep infection	Count	31	15	22	68
	% within CupGroup	1.1%	0.8%	1.7%	1.1%
	% of Total	0.5%	0.3%	0.4%	1.1%
Confusion	Count	15	2	2	19
	% within CupGroup	0.6%	0.1%	0.2%	0.3%
	% of Total	0.3%	0.0%	0.0%	0.3%
Chest related	Count	33	18	9	60
	% within CupGroup	1.2%	0.9%	0.7%	1.0%
	% of Total	0.6%	0.3%	0.2%	1.0%
Cellulitis	Count	4	5	3	12
	% within CupGroup	0.1%	0.3%	0.2%	0.2%
	% of Total	0.1%	0.1%	0.1%	0.2%
Cardiac arrhythmia	Count	12	5	2	19
	% within CupGroup	0.4%	0.3%	0.2%	0.3%
	% of Total	0.2%	0.1%	0.0%	0.3%
Acute renal failure	Count	11	9	0	20
	% within CupGroup	0.4%	0.5%	0.0%	0.3%
	% of Total	0.2%	0.2%	0.0%	0.3%
Acetabular implant loosening	Count	7	2	1	10
	% within CupGroup	0.3%	0.1%	0.1%	0.2%
	% of Total	0.1%	0.0%	0.0%	0.2%
Pyrexia	Count	1	3	3	7
	% within CupGroup	0.0%	0.2%	0.2%	0.1%
	% of Total	0.0%	0.1%	0.1%	0.1%
Fracture	Count	5	5	0	10
	% within CupGroup	0.2%	0.3%	0.0%	0.2%
	% of Total	0.1%	0.1%	0.0%	0.2%
Electrolyte imbalance	Count	9	1	2	12
	% within CupGroup	0.3%	0.1%	0.2%	0.2%
	% of Total	0.2%	0.0%	0.0%	0.2%
Cerebral vascular accident	Count	4	1	0	5
	% within CupGroup	0.1%	0.1%	0.0%	0.1%
	% of Total	0.1%	0.0%	0.0%	0.1%
Anemia	Count	7	3	1	11
	% within CupGroup	0.3%	0.2%	0.1%	0.2%
	% of Total	0.1%	0.1%	0.0%	0.2%
Others	Count	65	31	24	120
	% within CupGroup	2.4%	1.6%	1.8%	2.0%
	% of Total	1.1%	0.5%	0.4%	2.0%
No	Count	2252	1703	1151	5106
	% within CupGroup	83.5%	86.7%	87.2%	85.4%
	% of Total	37.7%	28.5%	19.2%	85.4%
	Count	2697	1964	1320	5981
	% within CupGroup	100.0%	100.0%	100.0%	100.0%
	% of Total	45.1%	32.8%	22.1%	100.0%

*Note*: *P* < 0.001 using crosstab function in SPSS with chi‐square test.

## Discussion

This study aimed to analyze which one of the multi‐factors could affect revision rates when the CPT has been combined with different cup implants and to help THR patients in the UK choose a better prosthesis combination. Thus, the variables of age, gender, BMI, diagnosis, surgeon grade, the material of cup implants, cup size and surgical approach were analyzed *via* cross tables, ANOVA and survival analysis. It is found that the CPT stem combined with the Trilogy cup showed the better characteristics in terms of survival trends on the revision ratios, Harris hip scores and complication ratios than other cups. Moreover, when the patient was female, the range of BMI was 18.5–29.9, the cup materials were stainless steel, the cup sizes were less than 28 mm, the surgeon was Trainee and the surgical approach was the Others not the Posterior or Anterolateral, the prognosis was better in survival analysis (*P* < 0.005).

### The Best Cup

Figures 4, 5, 6, 7 demonstrates that the line trend of the Continuum cup was the highest in the postoperative at 1 year and 5 years. The line trend of the Trilogy cup followed the Continuum cup closely, which means the Trilogy cup had good HHS results also. Nevertheless, part of the HHS, such as the pain score, was the subjective results of patients and may be impacted by the mental health of the patients,^16^ so this study also compared how the revision rates and the orthopedic‐related complications could affect the implant survival years.

According to the survival revision rate between cup groups within 12 years, Fig. 8 shows that the Continuum cup had the poorest outcome (*P* < 0.004). The median survival time of the Continuum cup was only 11 years which was lower than the Trilogy cup at 12 years. In complication ratios, the Trilogy cup is the best in orthopedic‐related‐case while the Continuum cup is the best in “No” complication ratios.

In short, a large part of THR patients who used the ZCA and the Continuum had the better one of the HHS and mHHS results. However, after synthesizing the results of the HHS, mHHS and the survival analysis results, the Trilogy cup has a better outcome of THR than the other cups. Therefore, when the CPT as the stem and combined with the Trilogy had a better prognosis than the other prostheses combinations.

### Better Prognosis Factors

However, this study not only focused on which implant combination is better but also explored which multi‐factors could affect the outcome of the combination. Figure 9 displays that the trends of the survival rate of age in the Trilogy within 12 years are similar, and also the median survival time (*P* = 0.294). Figure 10 shows that the gender trends had a huge difference, but the median survival time was similar. It is presented that females had a better outcome than males (*P* < 0.001). The reason for this result could be explained by Johnsen *et al*.^17^ that males have a higher risk of dislocation than females in a short‐term of postoperative THR. Figure 11 reports that the line trends of the 18.5 to 24.9 BMI group and the 25 to 29.9 BMI group were slightly higher than the other groups. Also, the median survival time of the BMI > =30 group only was 11.06 years, which was lower than the other groups (12 years) (*P* < 0.001). Sood *et al*.demonstrated that morbidly obese patients had a higher rate of complications in the postoperative time section.^18^ Also, weight gain will increase the risk of THR in the in the interim.^19^ The postoperative conditions of the underweight patients may be impacted by the nutritional intake, so the survival trends of the BMI < 18.5 group have a slight difference between the normal weight group and the overweight group. It was noted that the survival trends of the Trainee Surgeons had a better outcome than the Consultant (Fig. 12). The reason might be the patient's condition of the Trainee Surgeons were less complicated than the Consultant Surgeons. There was no evidence to prove that Trainees had worse postoperative outcomes than Consultants by comparing the survival years and revision rates in the existing literature.^20^

Figure 13 shows that stainless steel had the best postoperation results as the survival trend was higher than other groups (*P* < 0.001). Although research by Hwang *et al*. was related to a dual‐mobility cup, the results also indicate that the cup size was smaller and the rate of dislocation lower.^21^ This result was similar to the result shown in Fig. 14, where the outcome of the <28 mm was better (*P* < 0.001).

Figure 15 illustrates that the anterolateral and the posterior surgical approaches were not performed well as the Others Group in the post operation (*P* < 0.001). According to the case number of the THR patients that the anterior, Hardinge, lateral and modified hard surgical approaches consisted of the Others Group. Kwon *et al*.^22^ present that the rate of dislocation of the direct lateral approach was 0.43%, the anterolateral approach was 0.70%, but the posterior approach was 4.36% without soft tissue repair, and with a repair it was 1.01%. Therefore, the direct lateral approach has a better outcome than the other approaches.

### Strengths and Limitations

This study investigated a large cohort of THR with 5881 cases between October 1998 to September 2021 and included three major cups and associated demographical and clinical factors, for example, age, gender, BMI, revision ratios, hip scores, implant survival years, surgical level, approach ways, head size, etc. Thus, this research is reliable to provide clinical guidance and suggestions to improve the prognosis for THR patients.

However, this research also presented several limitations. For example, the Continuum cup had HHS data for 5 years, though its survival data covered 12 years. Even though the CPT combined with the Trilogy has a better outcome when comparing the multi‐factors, HHS, mHHS, revision rates and survival years, the price, rejection reaction, cup size, cup materials and surgical approaches should be further considered. For example, the materials of the cup implant, cobalt chrome, has a higher rate of wear resistance, ceramic can achieve wear resistance, hardness, strength and heat resistance, and stainless steel has low strength and ductility,^23^ but the postoperation of the Stainless‐Steel was better than other materials (Fig. 13). Thus, choosing the implant combination and how to complete the THR surgery were decided by patients and surgeons. In addition, this study has not investigated the surgical skills, for example, what the coverage rate of acetabular cup is in primary replacement, whether the acetabular cups are placed at the center of rotation of acetabulum. These questions should be studied in the future. In general, Consultants should consider their practical requirements for the THR patients, case by case.

Though this study included many factors, the coverage rate of acetabular cup in primary replacement and whether the acetabular cups are placed at the center of rotation of acetabulum have not been included, due to lack of the related data in our database. According to the literature, some have used such information in their studies. Ozden *et al*. reported that both acetabular cup position and initial coverage over acetabular cup less than 50% had no significant effect on cup survival.^24^ Mou *et al*. compared two surgical methods and found that one was better than another in the coverage rate, indicating that the higher the coverage rate, the better.^25^ Most previous studies agreed that a higher acetabular coverage rate would give lower revision,^26^, ^27^ although this topic is still to be further explored. There is little research reporting the relationship among the Trilogy, the coverage rates and revision rates. In terms of position for the implant, Hirakawa *et al*. suggested the so‐called best position of the femoral head center; also showed that hips with a lateral and superior cup position were revised, but a superior and medial position with a certain cup inclination angle did not need revision.^28^ However, there is no research on the relationship between the acetabular cup centre and Trilogy. In short, the coverage rate of acetabular cup and the position of acetabular cups are still to be explored in the future.

### Recommendation for Future Research

Although this study had a huge number of cases of THR patients, some of the cases had been done a long time ago, the earliest one in 1998. Given the fact that in the 21st century the progress of clinical medicine has been rapid and better methods in many aspects have been found, therefore, it is necessary to carry out similar research in the near future using the updated operation data and to analyze which multi‐factors could impact the revision in terms of cup types.

### Conclusion

This study aimed to compare three commonly used type of cups, ZCA, Continuum and Trilogy to investigate which cup would be better suited for CPT. The cohort included 5881 cases of THR with multi‐factors, demographically and clinically, and the three groups of data were analyzed and compared using cross tables, ANOVA and survival analysis. The results showed that that the CPT stem combined with the Trilogy cup gave the best characteristics in terms of survival trends on the revision ratios, Harris Hip Scores and complication ratios. In addition, given the combination that the THR patients were female, the range of BMI within 18.5–29.9, the cup materials as stainless steel, the cup sizes less than 28 mm and the surgical approach being not the posterior or anterolateral, the clinical outcomes were better than the other combinations. Nevertheless, it is our suggestion that the surgeon should consider their practical requirements for the THR patients, case by case.

## Authorship Declaration

All authors listed meet the authorship criteria according to the latest guidelines of the International Committee of Medical Journal Editors, and all authors are in agreement with the manuscript.

## Conflict of Interest Statement

The authors declare that there is no conflict of interest.

## Author Contributions

Yu Hong: Data analysis and paper original writing. Linda Johnston: Data resource. Weijie Wang: Conception, data analysis, paper review and writing, and supervision.
